# Complementarity and Area-Efficiency in the Prioritization of the Global Protected Area Network

**DOI:** 10.1371/journal.pone.0145231

**Published:** 2015-12-17

**Authors:** Peter Kullberg, Tuuli Toivonen, Federico Montesino Pouzols, Joona Lehtomäki, Enrico Di Minin, Atte Moilanen

**Affiliations:** 1 Department of Biosciences, University of Helsinki, Helsinki, Finland; 2 Department of Geosciences and Geography, University of Helsinki, Helsinki, Finland; 3 School of Life Sciences, University of Kwa-Zulu-Natal, Durban, South Africa; University of Colorado, UNITED STATES

## Abstract

Complementarity and cost-efficiency are widely used principles for protected area network design. Despite the wide use and robust theoretical underpinnings, their effects on the performance and patterns of priority areas are rarely studied in detail. Here we compare two approaches for identifying the management priority areas inside the global protected area network: 1) a scoring-based approach, used in recently published analysis and 2) a spatial prioritization method, which accounts for complementarity and area-efficiency. Using the same IUCN species distribution data the complementarity method found an equal-area set of priority areas with double the mean species ranges covered compared to the scoring-based approach. The complementarity set also had 72% more species with full ranges covered, and lacked any coverage only for half of the species compared to the scoring approach. Protected areas in our complementarity-based solution were on average smaller and geographically more scattered. The large difference between the two solutions highlights the need for critical thinking about the selected prioritization method. According to our analysis, accounting for complementarity and area-efficiency can lead to considerable improvements when setting management priorities for the global protected area network.

## Introduction

Complementarity and cost-efficiency are core concepts in spatial conservation prioritization and are widely adopted as fundamental design principles of protected area (PA) networks [1, 2]. Despite the thorough theoretical description [1–3], the effects of accounting for complementarity and cost-efficiency on the results of spatial conservation prioritization analyses are rarely discussed [4–9]. Does accounting for area-efficiency and complementarity increase the performance of the selected protected area network in terms of species representation and costs? What are their effects on the spatial arrangement of the top-priority areas? In this paper we reanalyze the results of a recent large scale conservation prioritization study of the global protected area network [10] using, unlike the original study, an approach that accounts for both complementarity and area-efficiency. We analyze the results in terms of difference in aggregate performance and spatial arrangement of the priority network.

The management effectiveness of protected areas influences how safe they are from anthropogenic pressure [11–14]. Despite political goodwill, there is extensive evidence that the global terrestrial environment continues to degrade both inside and outside protected areas [15], implying that management effectiveness is a serious concern. Le Saout et al. [10] responded to this concern by a study that provides policy recommendations on the management priorities within the global protected area network (hereafter GPAN). As one of the main results, their study identifies a set of 145 areas (5.5% of the total area of the GPAN), which are important for maintaining species globally and therefore should be designated as World heritage sites.

Le Saout et al. [10] use IUCN data on the ranges of terrestrial vertebrates to rank the protected areas according to their value for global biodiversity. They calculated conservation value separately for each protected area by summing up the transformed proportions of the species ranges occurring within its boundaries. A sigmoidal transformation was used to emphasize large range fragments over the small ones in order to reduce possible commission errors caused by inaccuracies in the data. Thus, the method gave high conservation value for protected areas that contained large proportions of ranges of many (narrow ranged) species. They call the measure irreplaceability and describe it as “an aggregated measure of the degree of dependence of species’ on the protected area” [10]. Effectively, it is a type of scoring method that does not explicitly account for complementarity or area-efficiency [16]. Therefore, top-priority areas could be relatively similar to each other, possibly over-emphasizing large species-rich tropical protected areas.

Accounting for protected area size relates to the spatial conservation prioritization concept of efficiency according to which it makes sense to achieve the same benefits with lower rather than higher resources (here area) [1, 17]. Alternatively, if operating on a fixed budget, one should aim for securing higher benefits while keeping resource use constant. Overall, accounting for costs has been a major theme in spatial conservation prioritization [2, 18, 19]. In the present case, the difference is between counting PAs [10] and accounting for area-efficiency which is about maximising the coverage of species ranges within a fixed area. An important point about efficiency is that it increases societal acceptability and that an efficient reserve system is more likely to succeed in the face of competing interests [20].

The other concept, complementarity, is about how well sites in a network work together in achieving conservation objectives [2, 3, 21]. One characteristic of a solution with high complementarity is that there is coverage for all (or most) biodiversity features, not just a small subset of them. Complementarity can be contrasted to scoring, which values an area primarily based on local considerations and which therefore does not guarantee that several sites work well together. The third key concept relevant for the present study is effectiveness. It is a more holistic concept than efficiency, and describes how well the aims of adequate representation and persistence of species and ecosystems are met [2, 19, 22]. Notably, effectiveness does not require efficiency [1]. Nevertheless, one could assume that the lack of efficiency and complementarity would in general reduce effectiveness, for example, because many species might be completely missed by a reserve network—an undesirable outcome from the perspective of conservation.

## Methods

We replicated the prioritization of protected areas by Le Saout et al. [10] using the Zonation conservation prioritization software, which produces a complementarity-based nested hierarchy of conservation priority through the landscape aiming to maximize conservation value per used resources (here area) [23]. This ranking is typically based on distributions of biodiversity features (here species ranges), distributions of costs, connectivity and other such factors. This approach has previously been applied to high-dimensional biodiversity data [24, 25], and the analysis is expected to produce solutions with a high return on (conservation) investment [26, 27]. For further information about Zonation see e.g. Moilanen et al. [28] and Lehtomäki & Moilanen [29].

We used the same data as in Le Saout et al. [10], barring small differences potentially arising from different access dates. We obtained species ranges for mammals, birds and amphibians from the IUCN Red List of Threatened Species [30] and information about the GPAN from the World Data Base on Protected Areas [31]. As in Le Saout et al. [10], we included all the PAs with explicit spatial polygon into the analysis. For use in Zonation, we approximated the original species range and protected area polygons by rasterizing them into 21 671 global high-resolution rasters (geodetic coordinate system, resolution 0.01667 degrees equaling 1.7 km at the Equator) with 9.7 million cells of information inside the GPAN. In some locations there are multiple PAs that spatially overlap with each other: such cases may arise when an area belongs to multiple conservation programs simultaneously. In these cases, we assigned raster cells to the protected area with the largest surface area, resulting in 93 837 distinct planning units. The set of 145 top-priority protected areas (137 designated and 8 proposed PAs) and two priority rankings lists identified by Le Saout et al. [10] were linked to spatial location, using the protected area ID number as the association key.

We did two different sets of Zonation analyses. In the first set, we adopted the same weighting scheme as in Pouzols et al. [32] and weighted species according to their IUCN threat status so that least concern (LC) species were assigned a weight of 1, near threatened (NT) and data deficient (DD) 2, vulnerable (VU) 4, endangered (EN) 6, and critically endangered (CR) 8. This approach implies that species persistence in the long run depends mostly on the GPAN which is an assumption justified at least in areas with high habitat loss rates. In the second set of analyses, we multiplied the threat status weights by the fraction of the range of the species that is inside the GPAN. This analysis gives higher emphasis on species that predominantly occur within the GPAN and are therefore highly dependent on it. We used PAs as planning units, meaning that although the species range polygons were approximated with high-resolution raster layers, the priority rank was defined at the level of protected areas. Additionally, we implemented two analyses in which top priorities were forced into large PAs. More specifically, we used 250 km^2^ and 1000 km^2^ as subjectively chosen minimum sizes for “large” PAs. We ran Zonation using the additive benefit function mode, which can be interpreted as the minimization of aggregate extinction risks via use of species-specific species-area curves [28].

To compare the fragmentation of species ranges between different solutions, we analyzed the size distribution of individual fragments of species ranges included in the top priority areas. This was done both in terms of area proportional to the total range and in absolute area. With range fragments we mean individual parts of species ranges that are formed by clipping the species ranges with the borders of top priority PAs. Technically, we obtained this information with the post-processing options available in Zonation [33] using the rasterized protected area layer that is described above.

## Results

**Fig 1:**
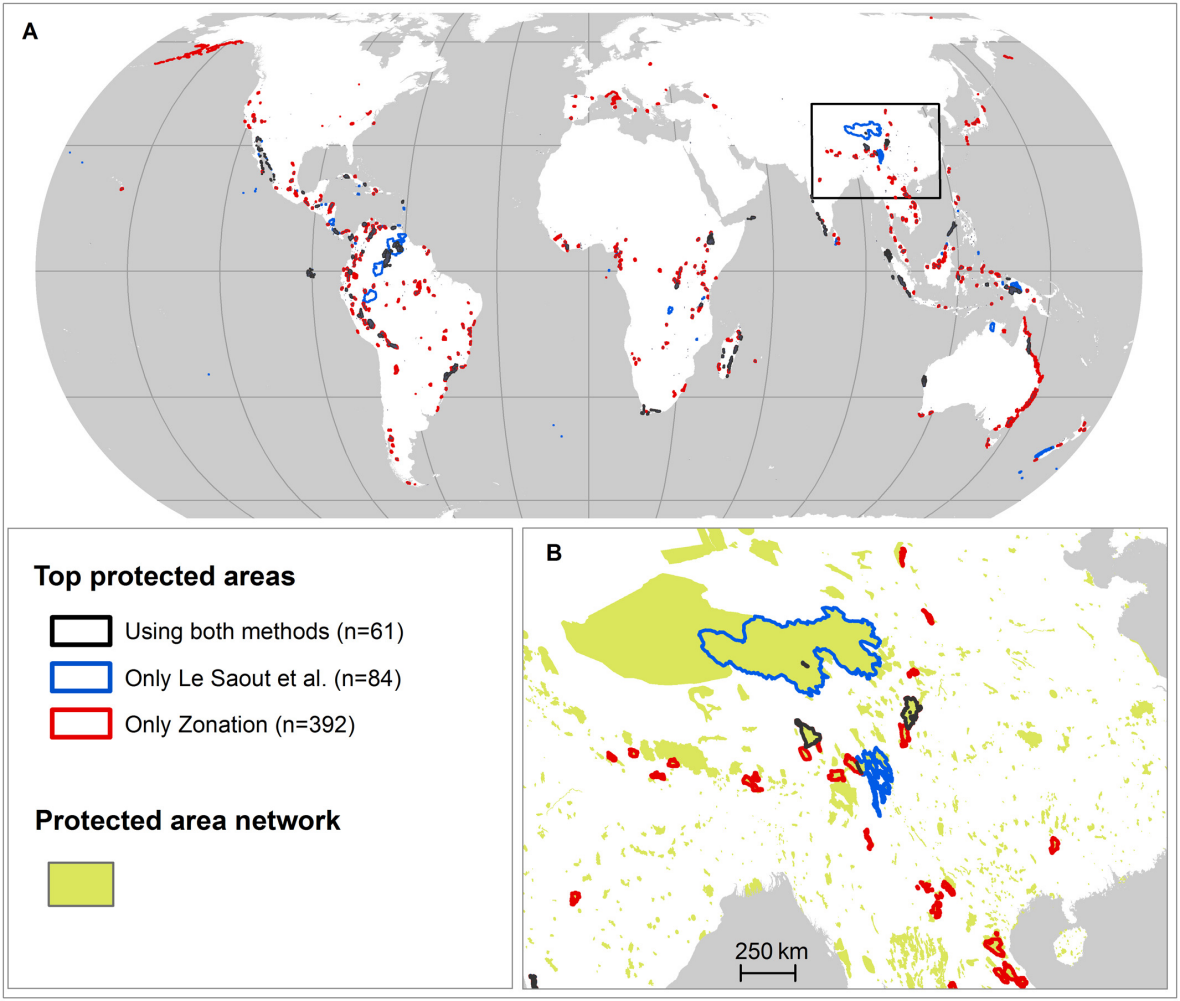
Spatial distribution of top priority areas. (A) Locations of the 145 top priority areas identified by Le Saout et al. [10] and the 453 top priority areas identified by the Zonation analysis when top priorities was limited to areas > 1000 km^2^ and species were weighted in proportion to their range within the GPAN. (B) Closer zoom to priority areas in Asia. The maps are in Eckert IV projection.

Fig 1 illustrates the different spatial priority patterns that arise from the different prioritization methods. While some priority areas overlap, the tendency is that the complementarity method gives higher priority to smaller areas than the scoring method. This is also reflected in the counts of areas selected by different analyses (Table 1) and by a relatively low overlap between the presently proposed priority areas and those of Le Saout et al. [10]. The global pattern of proposed priority areas is also different (Fig 1). The present analyses propose a greater global spread of areas, which is expected because accounting for complementarity promotes solutions that broadly cover the global species pool.

**Table 1:** Number, size and overlap of selected PAs in the alternative solutions.

Solution	N of Pas, excluding overlap	Mean size, excluding overlap (km^2^)	Shared area with 145 PAs of Le Saout et al. 2013 (%)
Priority ranking by Le Saout et. al. (2013)			
Combined	124	9740	100
All species	107	11329	91.5
Threatened species	253	4759	44.1
Zonation, species weighted according to their threat status			
PAs as PUs	7247	167	15.7
PAs > 250 km^2^	1267	954	17.3
PAs > 1000 km^2^	533	2267	23.6
Zonation, species weighted according to their threat status and proportional range within GPAN			
PAs as PUs	5871	206	22.7
PAs > 250 km^2^	1011	1194	27.3
PAs > 1000 km^2^	453	2667	30.3

All values are calculated for the top 5.5% of the GPAN, which corresponds to the total area of 145 priority sites proposed by Le Saout et al. [10] in their combined solution. Also included are statistics for two other priority rankings developed by Le Saout et al. [10]. Number and mean size of PAs are calculated using rasterized data layers where single cell can have only one value so that overlapping areas are excluded. Zonation results are given for analyses without limitations on size of PA, and for analyses where top priorities were limited to PAs greater than 250 or 1000 km^2^. Separate results are given for Zonation analyses that look at priorities in relation to global ranges of species or priorities in relation to ranges of species inside the GPAN only.

**Fig 2:**
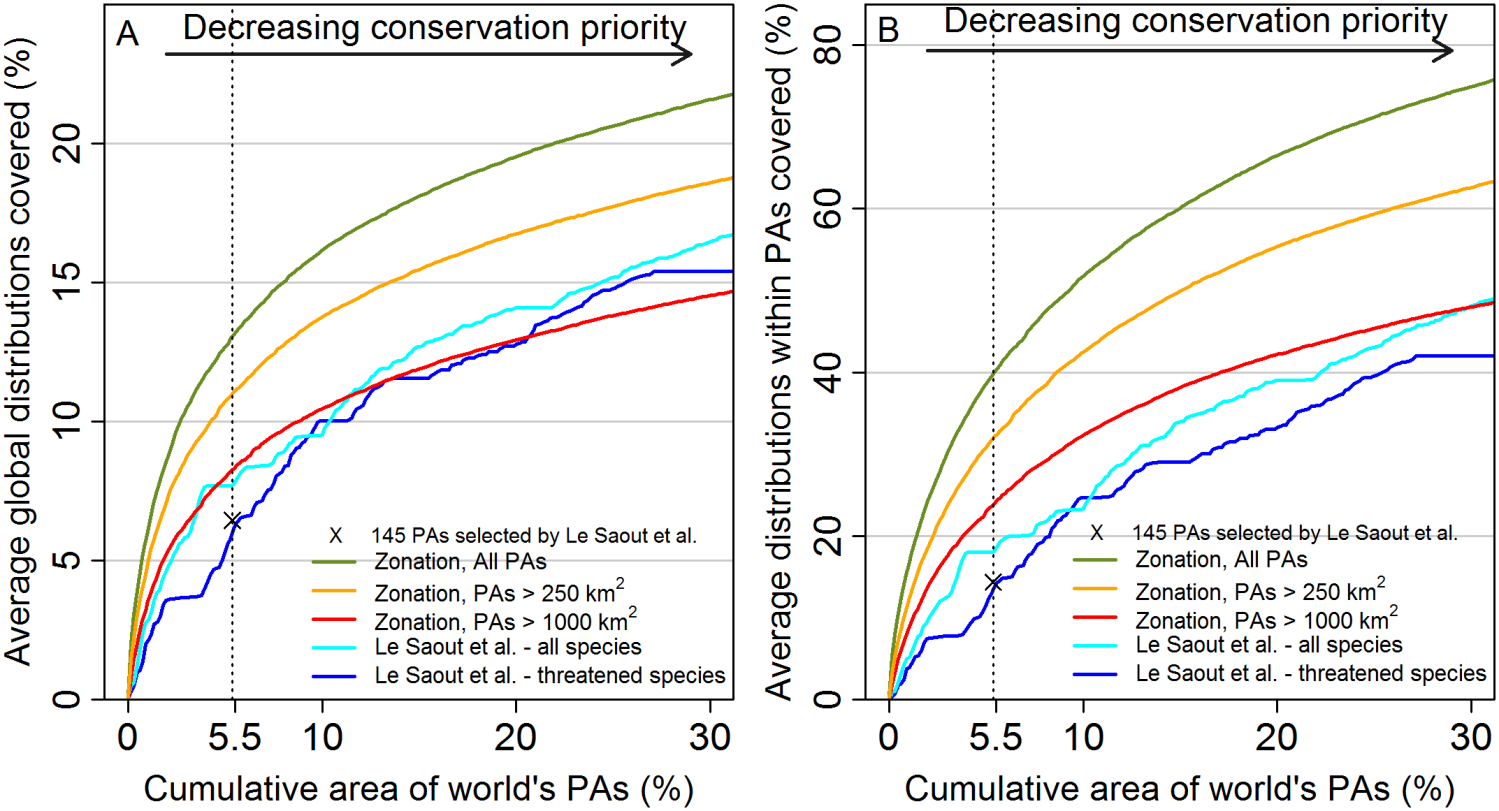
Performance curves for different selection methods, measured as the average proportion of species ranges covered. (A) Average performance of analysis variants with performance is measured in proportion to the global ranges of species. Here, species were weighted proportionally to the fraction of their global range within the GPAN, thereby accounting for the responsibility the GPAN holds for the species. (B) Performance of analysis variants, measured relative to range size inside the GPAN. Here, only the IUCN threat category influenced species weights. The vertical dotted line indicates the top fraction of PAs having the same joint size (5.5%) as the 145 PAs highlighted by of Le Saout et al. [10]. The performance of these sites is shown with a X mark on the dotted line. The mean range coverage at the 5.5% point can be used to compare the aggregate performance of different sets of top priority areas. Note different scales on the y-axis.

Fig 2 shows performance curves that are generated together with spatial priority maps such as the one shown in Fig 1. The performance curves show how much of the species ranges on average the selected PAs cover at any chosen top-priority fraction of the landscape. Here the point of interest is coverage of species at 5.5% of area of the GPAN, which corresponds to the total size of 145 PAs proposed by Le Saout et al. [10]. We show performance curves for three analyses, one of which has no constraints in terms of the size of PAs selected in the top 5.5%, the second constrains top areas to PAs greater than 250 km^2^ in size, and the third constrains top PAs to those greater than 1000 km^2^ in size. For comparison, we created similar performance curves for two priority rankings of Le Saout et al. [10] by forcing Zonation to select PAs in the order proposed by Le Saout et al. [10]. The highest coverage is always for the Zonation analysis with no size constraints followed by the size constrained analyses and areas proposed by Le Saout et al. [10].

**Fig 3:**
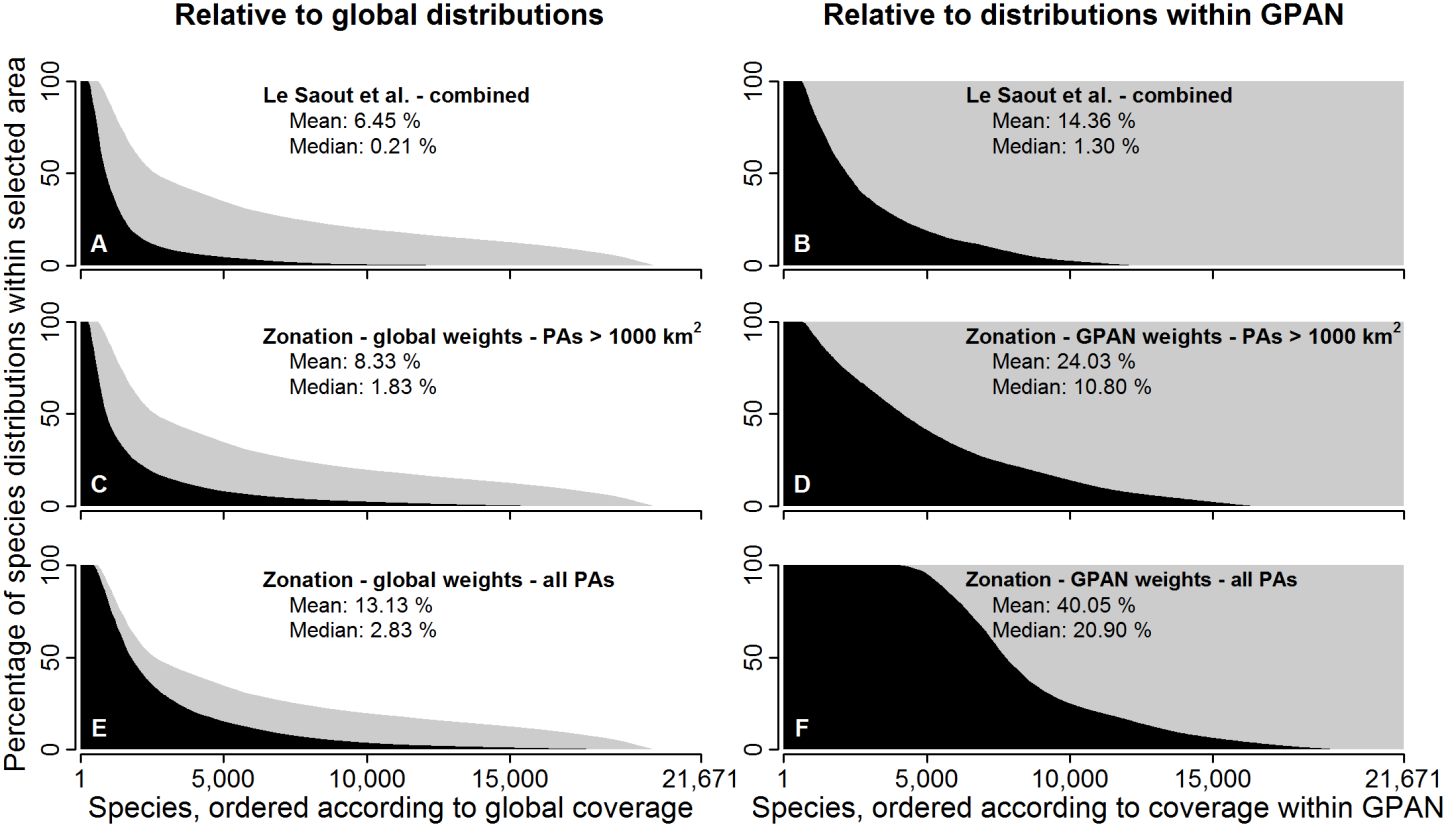
Coverage of IUCN species ranges in the top 5.5% of protected areas. In each panel the species are ordered from highest to lowest coverage relative to global range (left column) and range within PAs (right column). The gray shading represents the theoretical maximum achievable, which is defined by the proportion of species ranges inside the GPAN. Panels (A) and (B): 145 PAs selected using the method of Le Saout et al. [10]. (C) and (D): Top 5.5% of PAs prioritized with Zonation, including only PAs > 1000 km^2^. (E) and (F): PAs prioritized using Zonation without any protected area size restriction.

When comparing solutions, the averages shown in Fig 2 do not show how coverages vary across species. Going more into detail, Fig 3 shows the proportion of species ranges covered by the top 5.5% of the GPAN. Compared to the 145 PAs proposed by Le Saout et al. [10], the present complementarity-based analyses succeed in finding solutions that have up to 2.8 times the mean coverage of species ranges, up to six and a half times the number of species with full ranges inside top priority management areas, and only 30–70% the count of species lacking representation in the top areas. Table 2 shows these numbers in more detail. For example, comparing the 145 PAs identified by scoring method of Le Saout et al. [10] and the complementarity-based analysis (with no size limitation for priority PAs), one finds that the present results have double the mean range coverage when measured against global ranges, 14 times higher coverage for the median species, 72% more species with >99% range coverage within top priority areas, and half of the count of species with less than 1% of their ranges covered. When doing the analysis and comparison relative to ranges of species inside the GPAN, the differences are all around larger (Fig 3; Table 2). The relative performance of the Zonation analyses degrades when top priorities are restricted into very large PAs only, which is expected as constraints are placed on the solution (Table 2, Fig 3).

**Table 2:** Performance of alternative prioritization solutions, measured in terms of fractions of species ranges covered inside the top 5.5% of the GPAN.

	Percentage of species ranges within the top 5.5% of the GPAN compared to range inside the GPAN			Percentage of species ranges within the top 5.5% of the GPAN compared to global ranges of species		
	Mean %	Number of species		Mean %	Number of species	
Solution		> 99%	< 1%		> 99%	< 1%
Priority ranking by Le Saout et al. (2013)						
Combined	14.4	682	10576	6.4	298	13445
All species	13.6	624	10970	6.1	276	13732
Threatened only	18.1	914	8690	7.7	336	11694
Zonation, species weighted according to threat status						
PAs as PUs	40.0	4413	3381	12.5	456	6628
PAs > 250 km^2^	32.2	1389	4402	10.5	330	7356
PAs > 1000 km^2^	24.0	775	5993	7.9	255	8632
Zonation, species weighted proportionally to total range within GPAN and threat status						
PAs as PUs	37.8	3645	3550	13.1	513	6668
PAs > 250 km^2^	30.7	1373	4763	11.1	390	7660
PAs > 1000 km^2^	23.5	818	6551	8.3	294	8989

The solutions evaluated here are the same as explained in Table 1.

**Fig 4:**
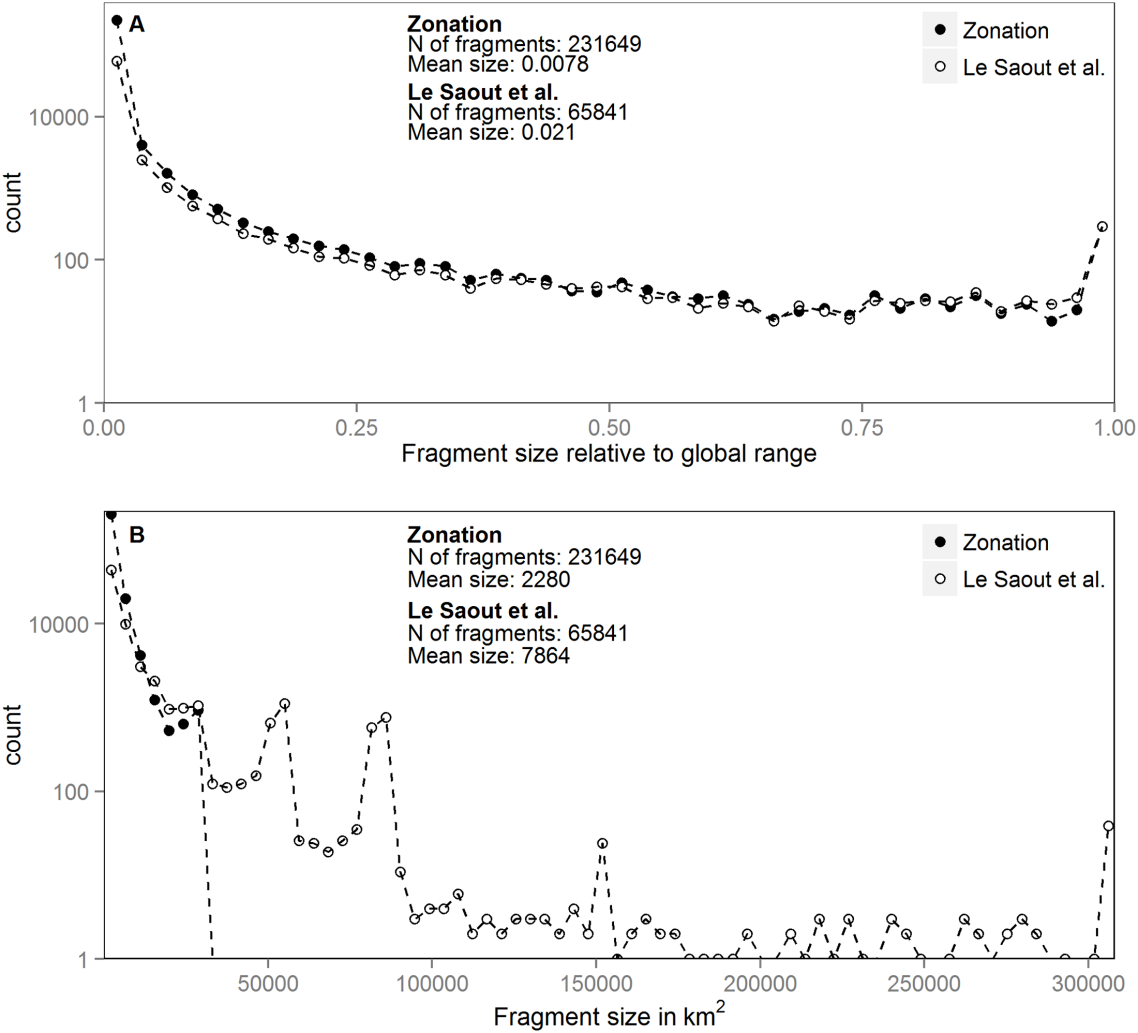
Size distribution of species range fragments in the top priority PAs. The plots compare the size distribution of species range fragments within two alternative sets of priority PAs: the Complementarity-based approach with a 1000 km^2^ size restriction and the one of Le Saout et al. [10]. Panel (A) summarizes the sizes of the fragments in relation to the global range sizes of species. Panel (B) describes the same information using absolute area (km^2^). Note logarithmic scale on y-axis.

The higher number of smaller protected areas and increased species coverage does not inevitably increase the fragmentation of the species ranges in the complementarity-based approach. This is seen in Fig 4 where the size distribution of fragments of species ranges within top priority sets of the scoring and the complementarity-based approach with 1000 km^2^ size restriction are examined. Fig 4A shows that when size is measured proportional to the total range of the species, both approaches include similar numbers of very large fragments that alone account for over 30% of the species distributions. Additionally, the complementarity-based approach includes large numbers of small fragments, which partly explains the increased species presentation but smaller average fragment size in the Zonation analyses. Overall, the number of small fragments compared to the large fragments is many orders of magnitude larger in both solutions. When the focus is turned into absolute area of the range fragments (Fig 4B) the situation changes. Individual range fragments can only be as large as the largest protected area in the solution. Therefore, the fragment size in the complementarity-based approach remains always under 30 000 km^2^ whereas in the solution of Le Saout et al. [10] fragment sizes ranges go up to 300 000 km^2^, according to the size of the largest protected area in the priority set. For fragments smaller than 30 000 km^2^ in area, counts of fragments is similar between solutions, although Zonation solutions include a higher frequency of smaller fragments.

## Discussion

We have reanalyzed the GPAN using the IUCN species range data in order to study the identification of management priority areas. We found that different assumptions make a great deal of difference to the outcome of conservation prioritization analyses: is complementarity accounted for or not; are costs (implicitly) represented by the PA count or by their joint area; is the context of analysis global ranges of species or alternatively the ranges of species inside the GPAN? Contrasting to a recently published scoring-based priority list [10], we were able to demonstrate noticeable improvements using a complementarity-based approach. Equally sized sets of areas that had almost three times the mean coverage across species, up to six and a half times the number of species with over 99% of the range in top priority areas and 1/3 the count of species with less than 1% of range inside the top priority areas.

What explains these differences? Both Le Saout et al. [10] and Zonation internally operate on the fraction of the range of a feature in a spatial unit. As such, both approaches could plausibly identify similar solutions. However, there are important differences that influenced the subsequent analysis. Zonation maximizes gains in relation to the size of the area whereas Le Saout et al. [10] calculated scores for areas without considering the size of the area leading to lower area-efficiency. As a second difference, Le Saout et al. [10] employ an increasing-by-area transformation at the level of individual PAs, thereby increasing the priority of large PAs. As further difference, Zonation includes a mechanism that tries to balance across features, thereby implementing complementarity. These differences lead to the differences in solution characteristics observed here (Figs 2 and 3, Tables 1 and 2).

The process of Le Saout et al. [10] favored very large PAs: the 145 areas chosen by them represented 5.5% of the area of the GPAN, which at the time of writing includes approximately 177 000 PAs. From these numbers it follows, that the top priority areas of Le Saout et al. [10] are approximately 85 times larger than the mean size of a PA. According to the present analysis, using complementarity-based selection and accounting for area-efficiency to select a slightly larger number of smaller areas would help represent a much higher fraction of species ranges in the top-priority areas. The present results also align with Cantu-Salazar & Gaston [11], who found that very large PAs generally include a low number of species and relatively few rare species. Nevertheless, focusing on small PAs can be a risky strategy as large PAs area generally considered to be safer against external pressures and better for species long term persistence [11, 34]. Setting a minimum size for the selected priority PAs or favoring large areas implicitly aims at a network that is more robust against external threats, but limiting the size has obvious tradeoffs. According to the present results, concentrating on very large PAs completely misses many species, if for no other reason that many species have their only known occurrences inside a small or medium-sized PA. As can be seen in Fig 3 there are hundreds of species that can only be covered in the top priority sets if selection of small areas is allowed.

It is important that even though the average size of protected areas is decreased in analyses that account for area-efficiency, this does not automatically mean a decrease in the number of large continuous fragments of species ranges within the top priority areas. First, the number of large (relative to range size) fragments goes up together with the amount of species ranges covered by the solution. Second, the area-efficient approach favors species with relatively narrow ranges because comparatively larger fractions of their ranges can be covered with any given area. Whether this is desirable depends on the aims of the analysis, but it has been argued that many of the species with high conservation importance have also small ranges [35, 36]. Even so, protection of for example large carnivores does require very large areas [37].

Here we have explicitly focused on area-efficiency in conservation prioritization, which can be interpreted as assuming equal per-area management cost over the whole study area. It would also have been possible to use data on estimated management cost and thus focusing on cost-efficiency [38]. We did not do so because, although we provide priority ranking of the PAs, our primary aim was not to point out areas for conservation action but to compare the effects of different methodological choices, and additional data would have complicated the comparison. Overall, to make prioritizations of the GPAN more informative for planning, further analyses could include more species groups and account for connectivity [29], multiple costs [28, 39] administrative division between countries [23] or account for changes in biodiversity patterns due to global change [40].

Although our analyses could be improved by including more data and factors relevant for planning, conservation prioritization analyses can never account for the full complexity of the real world. It is important to remember that conservation prioritization analyses are only a part of the whole planning process. More information and expertise is always needed in developing operational conservation plans [41]. Furthermore, we want to highlight that the most important outcome of this study is not the priority ranking itself but the way the assumptions, costs and benefits of different methodological choices are made explicit and quantified. This information is valuable for academic studies and real-life conservation plans alike.

Despite the better performance of complementarity-based approaches, such as the one we have shown here, scoring approaches are still widely used especially in practical applications because they are considered to be easier to develop, implement and describe [16]. Whether this conceptual simplicity is worth the potentially lowered species representation, has to be decided case-by-case. We believe that in most cases it is not. In any case, the decision should be done after explicit deliberation. The aims of the project and intended use of the priority products also define the suitability of different methods. Scoring can be seen as a method that does not make many assumptions about biodiversity in other sites, whereas the complementarity-based approach tries to identify a group of sites that work well together [16]. For example in the present case Le Saout et al. [10] aimed to identify a set of PAs that carry high responsibility for the persistence of global biodiversity. Scoring is appropriate for example when the focus is on evaluating the value of individual sites quickly, without possibility to account for the entire landscape. If the focus is to protect a representative sample of global biodiversity in a PA network, then accounting for complementarity is essential as it enables the identification of set of areas that cover biodiversity broadly and efficiently. Our view is that this would be a desirable feature also in a set of areas proposed as World Heritage Sites carrying high responsibility of protecting global biodiversity.

Well informed management and expansion of the present GPAN are of high importance when the world strives to maintain its ecosystems in response to the commonly agreed objectives of the Convention on Biological Diversity [42–44]. The present work demonstrates that making alternative analytical choices can lead to major differences in the proposed priorities. Therefore, conservation priorities should always be viewed as outcomes of a process involving methodological choices that, often implicitly, define how biodiversity is valued. There will be no ultimate priority product that would cover all aspects of conservation value and utility equally and it is up to whoever is doing a prioritization to assess whether a given method corresponds to given needs. Additionally, the societal context of PAs varies across the world [45, 46], implying that different approaches might be needed to better correspond to the socio-political realities of different countries.
